# Exposure to Gulf war illness-related chemicals exacerbates alcohol- induced liver damage in rodents

**DOI:** 10.21203/rs.3.rs-3838282/v1

**Published:** 2024-01-18

**Authors:** Anca Petrescu, Julie Venter, Dana D Danilenko, Daniela Medina, Stephanie Grant, Su Yeon An, Elaina Williams, Patrick Mireles, Matthew Tjahja, Sharon DeMorrow

**Affiliations:** The University of Texas at Austin; The University of Texas at Austin; The University of Texas at Austin; University of Pennsylvania; The University of Texas at Austin; The University of Texas at Austin; The University of Texas at Austin; The University of Texas at Austin; Baylor Scott & White Health; The University of Texas at Austin

## Abstract

Gulf War Illness (GWI) describes a series of symptoms suffered by veterans of the Gulf war consisting of cognitive, neurological and gastrointestinal dysfunctions. Two chemicals associated with GWI are the insecticide permethrin (PER) and the nerve gas prophylactic pyridostigmine-bromide (PB). In this study we assessed the effects of PER and PB exposure on pathology and subsequent alcohol (EtOH)-induced liver injury, and the influence of a macrophage depletor, PLX3397, on EtOH-induced liver damage in PER/PB- treated mice. Male C57BL/6 mice were injected daily with vehicle or PER/PB for 10 days, followed by 4 months recovery, then treatment with PLX3397 and a chronic-plus-single-binge EtOH challenge for 10 days. PER/PB exposure resulted in the protracted increase in liver transaminases in the serum and induced chronic low-level microvesicular steatosis and inflammation in GWI vs Naïve mice up to 4 months after cessation of exposure. Furthermore, prior exposure to PER/PB also resulted in exacerbated response to EtOH-induced liver injury, with enhanced steatosis, ductular reaction and fibrosis. The enhanced EtOH-induced liver damage in GWI-mice was attenuated by strategies designed to deplete macrophages in the liver. Taken together, these data suggest that exposure to GWI-related chemicals may alter the liver’s response to subsequent ethanol exposure.

## Introduction

Gulf War Illness (GWI) has been researched extensively not only due to its complexity, but also because more than 25% of the veterans deployed to the Persian Gulf during the 1990–1991 war, were affected by it^1^. GWI is defined as a multi-symptom syndrome characterized by neurological dysfunctions^2,3^, impaired memory and cognition^4^, chronic fatigue^5^, musculoskeletal pain^6,7^, gastrointestinal (GI) conditions^8^ and skin lesions^9^. These symptoms were associated with chronic low-grade increase of serum inflammation markers detected in Gulf War (GW) veterans^10-12^. The symptoms of GWI were characterized as being caused by various factors including dysregulation of the immune system and mitochondrial dysfunction^13^. Thus, increased systemic proinflammatory cytokines such as C-reactive protein and chemokine (C-C motif) ligand 2 (CCL2), were reported by clinical studies^14,15^, and more recently, certain positive acute phase proteins i.e. y-interferon-induced protein and serum amyloid A were associated with worse fatigue or pain in veterans meeting the GWI criteria ^16^. It was also demonstrated that veterans with GWI exhibited greater damage of mitochondrial DNA in peripheral blood mononuclear cells, as compared to healthy controls^13^.

Associated with the development of GWI is exposure to GW-related chemicals including pyridostigmine-bromide (PB), a prophylactic drug against nerve agents like sarin gas, and insecticides such as permethrin (PER) ^1,17^. While a direct association between GWI and a higher incidence of various liver diseases has not yet been suggested in patients, chronic exposure to low level amounts of these chemicals has previously been shown to cause mild toxicity and inflammation in the liver^18^ likely explained as a result of the xenobiotic metabolism of these agents in hepatocytes ^19^. It is conceivable, therefore, that these mild alterations in liver pathology as a result of exposure to GWI-related chemicals may alter the response and susceptibility to subsequent hepatotoxic insults. Indeed, we have previously demonstrated that exposure to GWI-related chemicals primed the hepatic proinflammatory response, and aggravated cholestasis-induced liver damage and fibrosis in rats^20^.

In the current study, we used a mouse model of GWI, to test the long-term effects of exposure to GWI-related chemicals on the susceptibility of the liver to subsequent chronic alcohol exposure. Furthermore, we used PLX3397 (Pexidartinib), an inhibitor of colony stimulating factor 1 receptor (CSF1R), to test the hypothesis that depletion of macrophages in the liver, could mitigate the liver injury caused by alcohol consumption in GWI-mice, as compared to negative controls (Naïve mice).

## Results

### GW-related chemicals PER and PB, increased serum transaminases and induced hepatic microvesicular steatosis in mice.

**Figure 1:**
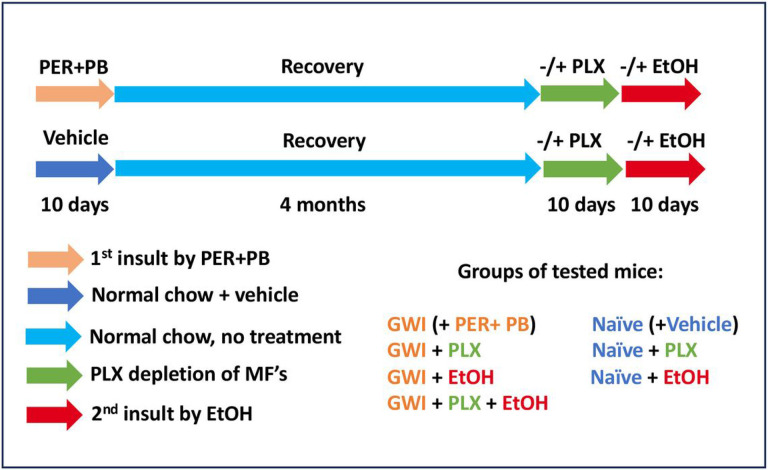
Timeline of in vivo experiments to assess the effects of GWI-related chemicals on mice exposed to PER and PB, vs Naïve mice. The additional treatments with PLX3397 inhibitor of CSF-1R in macrophages and alcohol (EtOH) were applied as shown in the diagram. Abbreviations: PER, permethrin; PB, pyridostigmine-bromide; EtOH, ethyl alcohol; PLX, PLX3397; MF, macrophages; GWI, Gulf War Illness.

**Figure 2:**
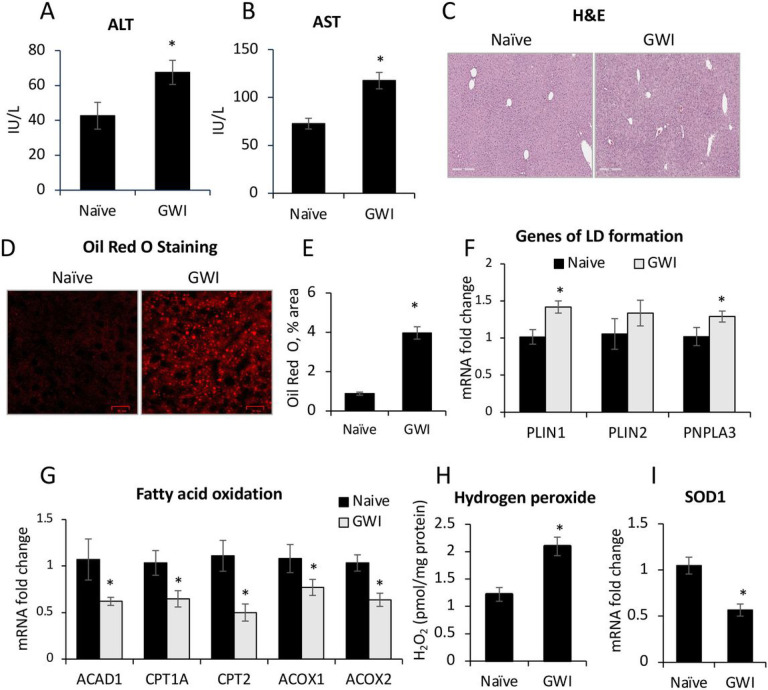
Exposure of mice to GWI-related chemicals caused chronic low-grade liver damage as detected by serum transaminases and microvesicular steatosis. ALT (A) and AST (B) transaminase levels in serum samples from Naïve and GWI-mice after 4 months of recovery from exposure to PER plus PB, or vehicle. (C), Images of H&E staining of liver sections from Naïve and GWI mice. (D), Images of lipid droplets detected by Oil Red O staining of liver sections, followed by confocal microscopy. (E), Image analysis and quantification of lipid droplets as percentage of stained area, in livers from Naïve and GWI mice. (F), RT-qPCR results for expression of genes associated with lipid droplets, i.e. *PLIN1, PLIN2* and *PNPLA3* relative to *GAPDH.* (G), RT-qPCR to test the expression of genes with role in fatty acid oxidation, i.e., *ACAD1, CPT1A, CPT2, ACOX1, ACOX2* in livers of Naive and GWI mice. (H), the level of hydrogen peroxide as a marker of reactive oxygen species (ROS), was determined in liver samples of GWI vs Naïve mice. (I), RT-qPCR results for *SOD1* gene that encodes for superoxide dismutase, a liver enzyme with role in elimination of ROS. N=4, p<0.05, *, GWI vs Naïve mice. Scale bar, 100 mm.

The model of GWI used in this study has previously been described and defined^21^ and was obtained by treating the mice with PER and PB for 10 days. In order to dissect the long term consequences of PER/PB treatment on the liver from the acute effects of PER/PB metabolism we allowed the mice to recover from PER/PB treatment for 4-months prior to the subsequent analysis of liver function as described under the Methods section. Mice treated with vehicle (Naive) on the same timeline as GWI-mice (Fig. 1), were used as negative controls. Serum transaminases alanine transaminase (ALT) and aspartate aminotransferase (AST) were slightly increased in GWI-mice as compared to Naïve controls (Fig. 2A, B), while the H&E histopathology test did not show differences between the two groups of mice (Fig. 2C), suggesting minimal overt liver damage due to PB/PER exposure itself. However, staining with Oil Red O demonstrated that livers of GWI-mice exhibited accumulation of fat in very small lipid droplets, a condition known as microvesicular steatosis, and image analysis indicated a significant increase in microvesicular steatosis in GWI-mice compared to Naïve controls (Fig. 2D, E). We assessed the expression of genes involved in lipid metabolism of the liver, and data indicated that the expression of perilipins 1 and 2 (PLIN1,2), which are structural proteins of lipid droplets, as well as the lipase PNPLA3, a marker of lipid droplets, were increased in GWI vs Naïve mice (Fig. 2F). Moreover, the expression of fatty acid oxidation genes encoding for acyl-coenzyme A dehydrogenase 1 (ACAD1), carnitine palmitoyltransferase 1A (CPT1A), (CPT2), acyl-CoA oxidase 1 (ACOX1), (ACOX2) were significantly reduced (Fig. 2G). These data suggested that GWI-related chemicals hindered lipid oxidation and induced lipid droplet formation in the liver. Furthermore, there was increased hydrogen peroxide and decreased superoxide dismutase 1 (SOD1) mRNA expression in the livers of GWI mice as compared to Naïve controls (Fig. 2H, I), suggesting that PER/PB exposure enhanced the level of reactive oxygen species (ROS) which are known to be associated with excess lipid accumulation in the liver^22^.

### PER/PB caused a low-grade but long-lasting increase in hepatic inflammation.

**Figure 3:**
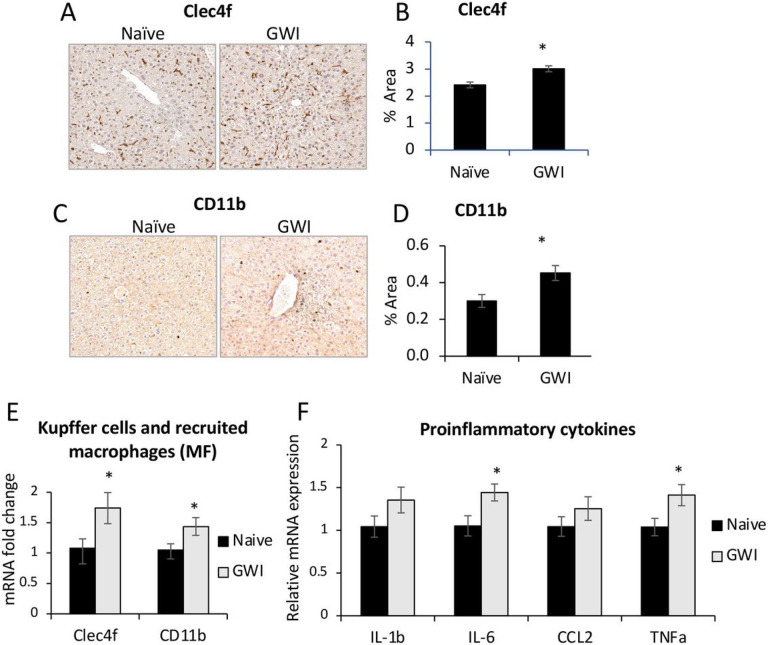
Exposure of mice to GWI-related chemicals resulted in chronic low-grade hepatic inflammation. (A), IHC images of Clec4f marker of Kupffer cells. (B), Quantification of CLEC4f of Kupffer cells as percent-stained area. (C), IHC of CD11b marker of infiltrated monocyte-derived macrophages, in liver sections of Naïve and GWI-mice. (D), Image analysis results for CD11b as percent-stained area, by IHC. (E), RT-qPCR for Clec4f and CD11b in livers of Naïve and GWI mice. (F), RT-qPCR results of proinflammatory genes *IL1b, IL6, CCL2 and TNFa* in livers of Naïve and GWI mice. N=4, p<0.05, *, GWI vs Naïve mice. Scale bar, 100 mm.

We have previously demonstrated an increase in inflammatory cell accumulation in the liver of a rat model of GWI^20^, to determine if this observation holds true in our mouse model of GWI, the density of Kupffer cells and monocyte-derived macrophages in livers of GWI and Naïve mice was assessed by immunolabeling these cells with specific antibodies to Clec4f and CD11b, respectively (Fig. 3). Exposure to PB/PER resulted in a significant increase in both types of macrophages as shown by pictures and quantification data in Fig. 3A, B for Clec4f and in Fig. 3C, D for CD11b. The mRNA levels of Clec4f and CD11b were also augmented in livers of GWI vs Naïve mice (Fig. 3E). Furthermore, several proinflammatory cytokines including IL-1b, IL-6, CCL2, TNFa were assessed, and data indicated similar significant increases in their expression (Fig. 3F).

### PER/PB induced low level, long-lasting fibrogenesis and ductular reaction in the liver.

**Figure 4:**
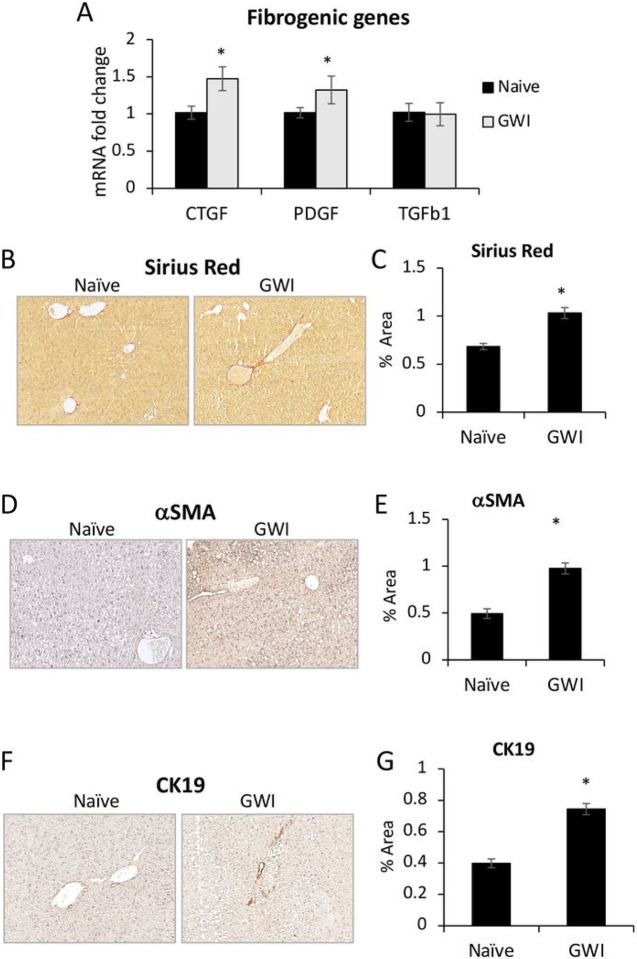
Exposure of mice to GWI-related chemicals PER and PB, induced chronic, low-grade hepatic fibrosis in mice. (A), Changes in mRNA expression of genes with role in fibrogenesis, i.e., *CTGF, PDGFb, TGFb1* in livers from Naïve and GWI mice. (B), Representative images of Sirius Red staining of liver sections. (C), Quantification of proteins that are markers of liver fibrosis, based on image analysis of Sirius Red staining in livers from Naïve and GWI mice. IHC images (D) and quantification by image analysis (E) was performed for aSMA protein that is a biomarker of activated HSC. The intrahepatic bile duct mass in livers of GWI vs Naïve mice, was measured by IHC of CK19 marker of cholangiocytes; representative images (F) and quantification (G) are shown. N=4, p<0.05, *, GWI vs Naïve mice. Scale bar, 100 mm.

We further investigated the effects of PER/PB on hepatic fibrosis. The expression of genes related to fibrogenesis, i.e., connective tissue growth factor (CTGF), platelet-derived growth factor-beta (PDGFb) and tumor growth factor beta 1 (TGFb1) was measured and found to be increased for CTGF and PDGFb in livers from GWI mice compared to Naïve controls (Fig. 4A). Sirius Red staining of collagen I and III showed a small but significant increase in these components of extracellular matrix (ECM) in GWI mice (Fig. 4B, C). A marker of hepatic stellate cell (HSC) activation is alpha-smooth muscle actin (aSMA) and it was shown to be increased in GWI mice vs Naïve mice by IHC staining of the liver sections (Fig. 4D, E).

Based on reported data^23^, hepatic fibrosis is frequently associated with ductular reaction, i.e., excess proliferation of cholangiocytes. Therefore, we measured the level of a cholangiocyte marker, cytokeratin 19 (CK19) and found that more CK19 was present in liver sections of GWI mice compared to Naïve controls (Fig. 4F, G).

### The effects of PER/PB on HepG2 cells in vitro.

**Figure 5:**
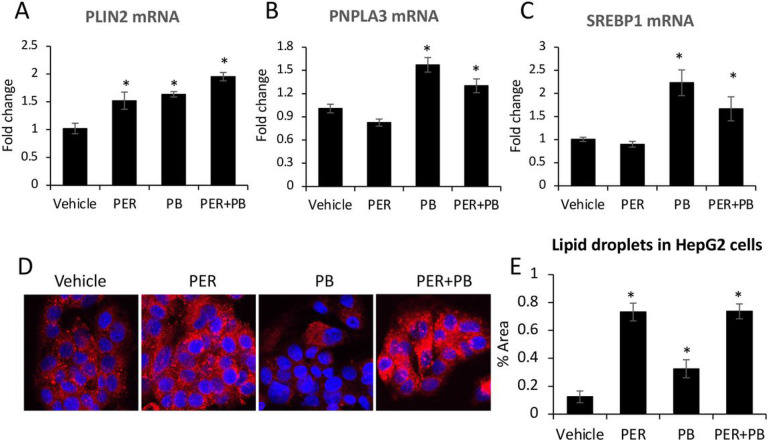
The effects of PER and PB on HepG2 cells in vitro. HepG2 cells were treated with PER, PB or a combination of PER and PB as described under Methods. Changes induced by these treatments in mRNA’s encoding for PLIN2 (A), PNPLA3 (B) and SREBP1 (C), were measured using RT-qPCR. The influence of PER, PB or PER+PB on lipid accumulation in HepG2 cells, was assessed by staining of the lipids with Nile Red: images of Nile Red-labeled lipid droplets (D) and quantification of area percent staining (E) are shown. N=4, p<0.05.*PER, PB, PER+PB vs Vehicle. Scale bar, 100 mm.

To test whether PER and PB exposure interferes with lipid metabolism in hepatocytes in vitro, we treated HepG2 cells with PER only, PB only, or a combination of both, as described under the Methods. The expression of lipid droplets-related genes such as PLIN2 and PNPLA3 at mRNA level, was assessed and the data indicated that: i) PLIN2 was upregulated by each substance individually, and even more by the combination of both PER/PB (Fig. 5A); ii) PNPLA3 was upregulated by PB only either individually or in combination with PER (Fig. 5B). To test the possibility that the lipids accumulated into hepatic lipid droplets were products of *de novo synthesis* of fatty acids, we measured the changes in mRNA of sterol regulatory element binding protein 1 (SREBP1), a transcription factor that upregulates genes of lipid synthesis^24^. As shown in Fig. 5C, PB only increased SREBP1 mRNA when administered alone or in combination with PER. These data suggested that PB stimulated *de novo synthesis* of lipids while PER upregulated proteins with role in lipid droplet formation.

The effect of PER, PB and their combination on lipid droplet formation in HepG2 cells was assessed by confocal microscopy imaging of Nile-Red-stained cells as described under the Methods (Fig. 5D, E). While PB had a small stimulatory effect on lipid accumulation into HepG2 cells, PER increased lipid droplets the most when alone or in combination with PB. Taken together, these data suggest that the effects of exposure to GWI related chemicals observed in vivo and likely due to the direct effects of these chemicals on the liver, rather than as an indirect result of their metabolism elsewhere in the body.

### Pre-exposed to PER/PB exacerbated subsequent EtOH-induced liver injury.

**Figure 6:**
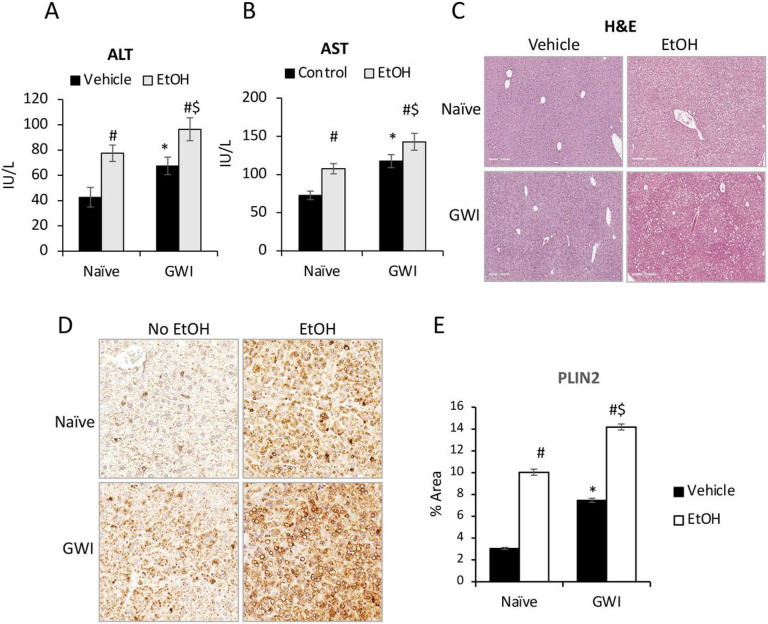
The effects of ethanol (EtOH) binging on liver function in GWI mice vs Naïve mice. ALT (A) and AST (B) transaminases were measured in serum samples from Naïve and GWI mice when treated with EtOH vs negative control mice. (C), H&E staining of liver sections were observed for histopathology and steatosis. (D), Images of PLIN2 immunostaining in liver samples from Naïve/GWI mice that binged on EtOH vs mice that did not receive EtOH. (E), Image analysis data for PLIN2 IHC in the mice described for panel D. N=4, p<0.05.*GWI vs Naïve mice. #, EtOH vs no EtOH within same group of Naïve or GWI mice. $, GWI+ EtOH vs Naïve+ EtOH. Scale bar, 100 mm.

To determine if these changes in the liver steatosis and inflammation due to exposure to GWI-related chemicals resulted in functional differences and susceptibility to a “second hit”, we treated Naïve and GWI-mice with EtOH or control diets for 10 days after 4 months of recovery from GW-related chemical exposure. The serum transaminases were increased due to EtOH consumption in both groups of Naïve and GWI-mice, however, there was a higher increase in EtOH-consuming GWI-mice compared EtOH-consuming Naïve mice (Fig. 6A, B). H&E staining of liver sections revealed that there was visible steatosis after EtOH treatment in both GWI- and Naïve-mice and (Fig. 6C). To quantify the level of steatosis, PLIN2, a protein associated with lipid droplets, was assessed by IHC in livers from Naïve and GWI mice that had been exposed to EtOH vs no EtOH (Fig. 6D, E). As shown by the IHC images and quantification of the immuno-stained percent area, the level of hepatic PLIN2 was dramatically increased in Naïve and GWI mice that consumed EtOH vs control mice. The amount of PLIN2 was more abundant in GWI mice vs Naïve mice that binged on EtOH.

**Figure 7:**
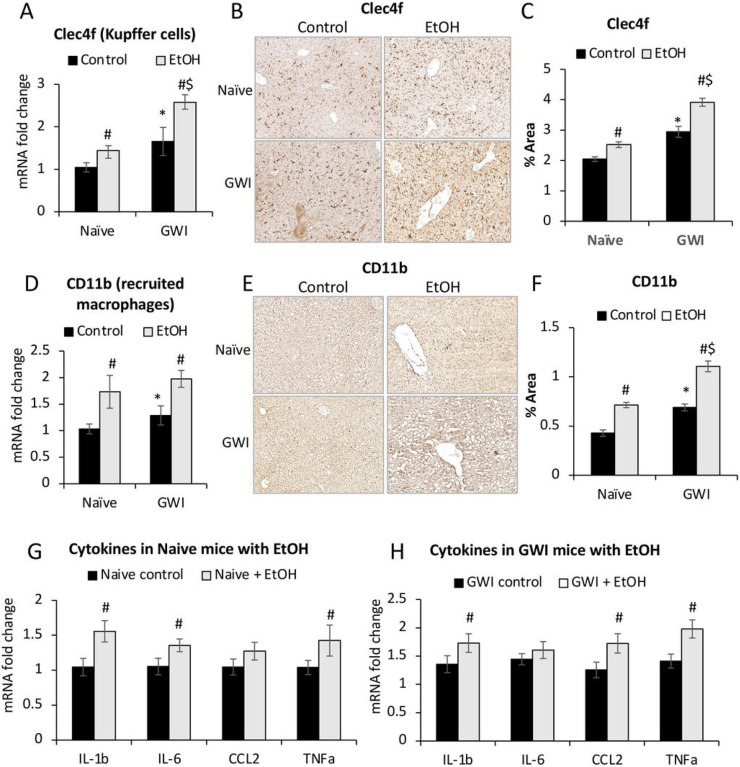
The effects of EtOH on hepatic inflammation in GWI-mice vs Naïve mice. The density of Kupffer cells was assessed by measuring *Celc4f* expression at mRNA (A) and protein (B, C). Changes in mRNA (D), and protein (E, F) levels of *CD11b.* Changes in mRNA of genes with role in inflammation (*IL1b, IL6, CCL2, TNFa*) were determined using RT-qPCR for livers from Naive mice (G) vs GWI mice (H) in the absence or presence of EtOH. N=4, p<0.05.*GWI vs Naïve mice. #, EtOH vs no EtOH within same group of Naïve or GWI mice. $, GWI+ EtOH vs Naïve+ EtOH. Scale bar, 100 mm.

Further measurements of hepatic inflammation markers were carried out to assess the effect of EtOH consumption on pro-inflammatory macrophages. The expression of mRNA and protein of Clec4f, which is specific to Kupffer cells, was found to be increased by EtOH in both groups of GWI- and Naïve-mice, and the highest level of expression was in GWI-mice that consumed EtOH (Fig. 7A, B, C). The CD11b marker of recruited monocytes-derived macrophages was also increased in EtOH-treated GWI- and Naïve-mice, with higher level in GWI-mice vs Naïve controls (Fig. 7D, E, F).

The effect of EtOH binging on proinflammatory cytokines in livers of Naïve (Fig. 7G) and GWI mice (Fig. 7H) was also tested and indicated that EtOH consumption caused increase in the expression of IL-1b, IL-6, CCL2 or TNFa in both groups of mice. However, some differences in mRNA levels, e.g., for TNFa and IL-1b, were higher in GWI mice compared to Naïve control mice. Moreover, while in the Naïve mice, EtOH binging did not change CCL2 mRNA levels; in GWI mice, there was a significant upregulation of *CCL2* gene induced by EtOH consumption.

### PER/PB exposure enhanced liver fibrosis and ductular reaction caused by EtOH consumption in mice.

**Figure 8:**
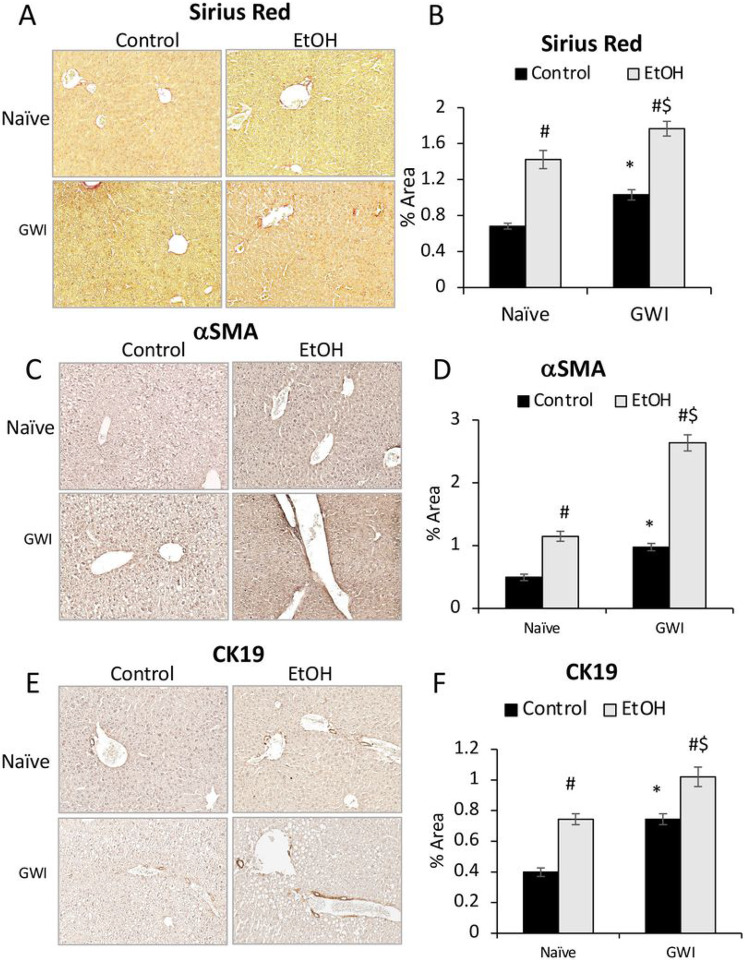
Influence of EtOH on liver fibrosis and intrahepatic biliary duct mass in GWI-mice vs. Naïve controls. Liver sections from GWI and Naïve mice that consumed EtOH vs mice that didn’t consume EtOH, were assessed for fibrosis using Sirius Red staining of ECM proteins and aSMA IHC. (A), Representative images of Sirius Red-staining. (B), Quantification of % area stained by image analysis. Images of aSMA IHC, and quantification data based on Image analysis are shown in panels C and D, respectively. Changes in IBDM were assessed by CK19 IHC: E, representative images; (F), quantification of CK19 based on image analysis. N=4, p<0.05.*GWI vs Naïve mice. #, EtOH vs no EtOH within same group of Naïve or GWI mice. $, GWI+ EtOH vs Naïve+ EtOH. Scale bar, 100 mm.

The effects of EtOH on liver fibrosis in GWI-mice vs Naïve controls were assessed using Sirius Red staining of ECM proteins in liver sections and showed that EtOH induced significant increase in these proteins in livers from Naïve and GWI-mice, with a slightly larger expression in GWI-mice vs controls (Fig. 8A, B). The marker for activated HSC, aSMA, was also assessed and indicated that EtOH consumption resulted in upregulation of aSMA in both Naïve and GWI-groups, with a larger increase of aSMA in GWI-mice vs Naïve controls (Fig. 8C, D).

The effect of EtOH on intrahepatic bile duct mass (IBDM) was measured, and all EtOH-consuming mice exhibited increased IBDM as compared to mice that had not been treated with EtOH (Fig. 8E, F). However, the EtOH-treated mice which were pre-exposed to GWI-related chemicals had higher IBDM than Naïve mice.

### PLX3397 treatment prior to EtOH consumption attenuated the EtOH-induced liver inflammation in mice pre-exposed to PER/PB.

**Figure 9:**
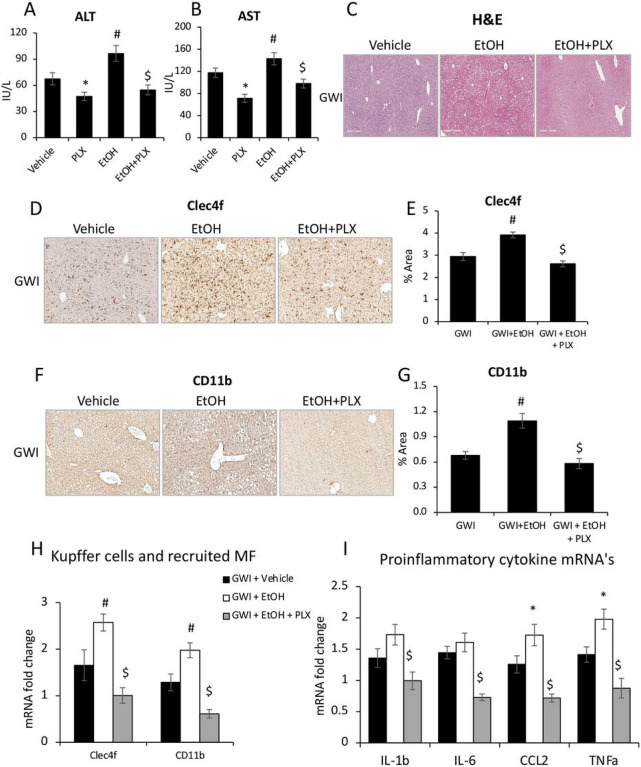
Depletion of macrophages improved liver function, and reduced hepatic inflammation caused by EtOH consumption in GWI mice. Serum transaminases ALT (A) and AST (B), as well as images of H&E staining (C) in Naïve or GWI mice that were treated with vehicle, or PLX3397 before EtOH binging (i.e., PLX, +EtOH), are shown. IHC images of Clec4f marker of Kupffer cells, and quantification data using image analysis are illustrated in panels (D) and (E), respectively. CD11b marker of infiltrated monocyte-derived macrophages in the liver was measured by IHC (F) and quantification using image analysis (G). Changes in Clec4f and CD11b mRNA were measured in livers from GWI mice when treated with vehicle, or PLX3397 prior to EtOH (H). Relative expression of mRNA for IL-1b, IL-6, CCL2 and TNFa was measured in the same groups of mice (I). N=4, p<0.05.*PLX vs vehicle. #, EtOH vs vehicle. $, PLX+ EtOH vs EtOH. Scale bar, 100 mm.

We investigated the possibility of using PLX3397 (shortly, PLX), a drug that was previously shown to neutralize macrophage activity^25^, to prevent the EtOH-caused pro-inflammatory response in mice that were exposed to GWI-related chemicals. The following groups of GWI-mice were analyzed: i) GWI exposure alone; ii) GWI + EtOH were treated with ETOH in the absence of PLX; iii) GWI + PLX were treated with PLX only; and iv) GWI + EtOH + PLX were treated with PLX prior to EtOH binging as described in the Methods. The level of serum transaminases indicated that PLX had a beneficial effect on the liver of GWI-mice, significantly reducing ALT and AST in GWI mice that were not exposed to EtOH as well as in GWI-mice that consumed EtOH (Fig. 9A, B). The H&E staining showed increased steatosis and hepatocyte distress especially in GWI-mice that consumed EtOH, and this type of damage was not observed in GWI-mice that were treated with PLX prior to EtOH binging (Fig. 9C).

The measurements of Clec4f by IHC, indicated that the EtOH-induced increase in Kupffer cells was prevented in GWI-mice that received PLX prior to EtOH consumption (Fig. 9D, E). Similarly, PLX treatment prevented the EtOH-induced increase in recruited monocyte-derived macrophages (Fig. 9F, G). The expression of *Clec4f* and *CD11b* genes at mRNA level also suggested that PLX downregulated the transcription of these genes of liver macrophages (Fig. 9H). Additional data on the influence of PLX on the expression levels of *Clec4f, CD68, F4/80* and *CD11b* mRNA’s in Naïve and GWI-mice treated with vehicle or PLX, are presented in supplementary Fig. 2A, indicating that except for F4/80 marker of macrophages, all the others were increased in GWI-mice and were reversed to normal or even to lower levels, by PLX. Moreover, *Clec4f* and *CD68* mRNA’s were downregulated as result of PLX treatment of Naïve mice as well (Suppl. Figure 1A).

The assessment of mRNA’s encoding for pro-inflammatory cytokines such as IL-1b, IL-6, CCL2 and TNFa indicated that these were upregulated by EtOH in GWI mice and downregulated in livers of GWI mice that were treated with PLX prior to EtOH consumption (Fig. 9I). Additional data on the expression of genes encoding for these cytokines in Naïve mice treated with vehicle or PLX, are shown in supplementary Fig. 1B. These results indicated that PLX did not change the mRNA levels in Naïve mice, while the upregulated levels in livers from GWI mice were reduced to normal in GWI mice that received PLX (Suppl. Figure 1B). The effect of PLX treatment on CD68-expressing macrophages in Naïve and GWI mice, was also measured (Suppl. Figure 1C, D). The data indicated that the increase in CD68 in the liver, which was caused by PER/PB exposure of GWI-mice, was reversed by PLX treatment (Suppl. Figure 1C, D).

### PLX3397 attenuated the EtOH-induced biliary hyperplasia and liver fibrosis in mice pre-exposed to PER/PB.

**Figure 10:**
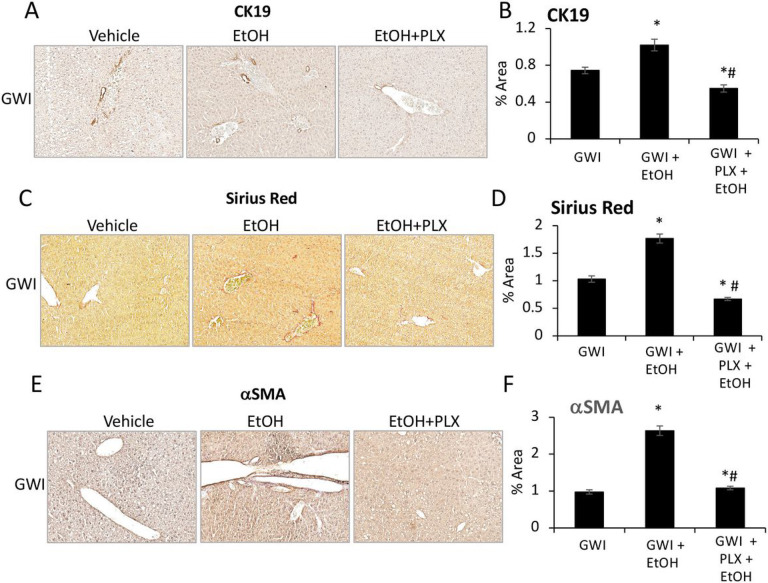
PLX3397 mitigated the increase in ductular reaction, fibrosis and ER stress in the liver of GWI mice binging on EtOH. (A), Representative images of CK19 IHC in livers of GWI mice treated with vehicle, EtOH or EtOH+PLX. (B), Image analysis quantification of CK19 IHC. Markers of fibrosis such as collagens and other ECM proteins were stained with Sirius Red: images (C) and quantification (D) are shown. aSMA, as indicator of HSC activation, was tested by IHC; images and quantification of aSMA IHC are shown in panels (E) and (F), respectively. N=4, p<0.05.*EtOH vs vehicle. #, EtOH vs PLX+ EtOH. Scale bar, 100 mm.

The assessment of IBDM using IHC for CK19 in liver sections, indicated that the increase in IBDM due EtOH, was prevented in GWI mice that were treated with PLX prior to consuming EtOH (Fig. 10A, B). Markers of fibrosis such as Sirius Red staining (Fig. 10C, D) and aSMA (Fig. 10E, F) which were elevated in GWI + EtOH mice vs GWI mice, were found to be lower in GWI + EtOH + PLX mice compared to GWI + EtOH mice. Moreover, Sirius Red staining indicated less ECM proteins in GWI + EtOH + PLX mice vs GWI-mice with no EtOH binging. The expression levels of genes with role in hepatic fibrogenesis, encoding for TGFb1 and CTGF were also determined (Suppl. Figure 2). Thus, Tgfb1 mRNA was not affected by GWI-related chemicals in the absence or presence of PLX, however it was highly upregulated as result of EtOH consumption in both groups of Naïve and GWI mice, with a larger increase in GWI mice that binged on EtOH. CTGF mRNA was upregulated in GWI vs Naïve mice, and ETOH binging enhanced this upregulation (Suppl. Figure 2). Treatment of GWI mice with PLX prior to EtOH, mitigated the effect of EtOH on expression of TGFb1 and CTGF.

## Discussion

In this study, we used a mouse model of GWI to investigate several aspects of liver inflammation and injury in GWI vs Naïve mice. Thus, we addressed two main aspects: i) a possible negative influence of PER and PB, on the liver; ii) exposure to GWI substances could prime the liver to over-react to a second insult such as EtOH binging.

Our first hypothesis, that exposure to a combination of PER and PB could result in a chronic low-grade inflammation of the liver, was based on several reports which indicated that in rodent models of GWI as well as in veterans manifesting symptoms of this syndrome, it was found a long lasting, higher than normal level of pro-inflammatory cytokines in the serum^10,11,14^. The primary focus of many publications on GWI studies are on the effects of this systemic low-grade inflammation in the central nervous system (CNS), because the most prevalent symptoms of veterans suffering from GWI were related to neurological diseases^26-28^. However, more recent reports suggest that disorders of CNS, as well as dysregulation of peripheral nervous system that controls the GI organs, were associated with exposure to substances used in the GW^29^.

The hypothesis that exposure to PER and PB could prime the liver to become more vulnerable and suffer increased damage when subjected to a subsequent insult in the form of alcohol-induced liver damage, was based on our prior observations of cholestasis in a GWI model in rats. As previously described^20^, we demonstrated that the exposure of rats to PER and PB followed by a period of recovery had a significant impact, enhancing the level of hepatic inflammation and damage caused by bile duct ligation (BDL)-induced biliary cholestasis. Therefore, in the current study, we tested whether chronic low-grade inflammation caused by PER and PB could affect the process of liver healing after a secondary insult produced by alcohol binging. Indeed, the results showed significant increase in liver damage after alcohol binging in GWI-mice, as compared to Naïve mice, as indicated by more acute inflammation, steatosis, ER stress and fibrosis. When compared to the rat model, the mouse GWI model showed a slightly higher level of pro-inflammatory indicators after PER/PB treatment even without EtOH treatment. Thus, in these mice, pro-inflammatory cytokines IL-1b, IL-6 and TNFa remained upregulated in the livers of GWI-mice even after a long period of recovery post GW-chemical exposure, suggesting a priming of the liver for acute response in case of a subsequent injury. Further investigation of HSC activation, bile duct proliferation and fibrosis concluded that all these symptoms of liver damage, caused by alcohol binging, were more severe in mice that had been exposed to GW substances, compared to Naive mice. It is worth mentioning that alcohol binging by Naïve mice resulted also in steatosis, liver inflammation, ductular reaction and HSC activation, however, the levels of these were lower compared to GWI-mice.

Regarding possible toxic effects of PER on the liver and other organs, a review was published in 1994 (NCBI Bookshelf ID: NBK231573, National Research Council (US) Subcommittee to Review Permethrin Toxicity from Military Uniforms. Health Effects of Permethrin-Impregnated Army Battle-Dress Uniforms. Washington (DC): National Academies Press (US); 1994; https://www.ncbi.nlm.nih.gov/books/NBK231573/?report=printable ). The review found that the only organ affected by PER in animal toxicity trials was the liver. Thus, doses equal or larger than 100 mg/kg PER ingested by rats for 26 weeks resulted in increase in liver weight and hepatocellular hypertrophy, characterized by an enlarged endoplasmic reticulum associated with increased activity of cytochrome-P-450-enzymes. The review concluded that the daily exposure to PER-impregnated uniforms at a level of 6.8x10^−5^ mg/kg/day, the toxicity that might come from wearing PER-impregnated uniforms should not be a concern. However, numerous reports on clinical studies in the following years, pointed out that an increasing number of veterans, up to 25% of those who participated in the GW, were affected by GWI and received treatments for GWI symptoms^30^. Also, additional studies on cytotoxic effects of PER on mouse liver cells reported that PER caused severe damages including proliferation of Kupffer cells, decreased nuclear size and increased vacuoles in hepatocytes^31^. Since PER is the most used insecticide not only in the military but also in agriculture and population disease control, the studies on its safety have been run with considerable scrutiny. Thus, a study that addressed the possibility that PER could affect differently people depending on their age, concluded that at least in animal experiments, exposure to PER at early age had more signs of liver damage such as dysregulated redox system and increased lipid peroxidation, as compared to adult controls^32^. A risk of increased incidence of liver cancer due to long term exposure to PER was also reported, showing that chronic exposure to PER increased the incidents of hepatocellular adenoma in female mice at a concentration levels of 2500 ppm or higher as compared to controls^33^.

Our experiments revealed that a relatively short-term exposure of mice to PER and PB, resulted in hepatic microvesicular steatosis which was persistent even after a long period of recovery. Also, in vitro experiments showed that HepG2 cells exhibited a significant increase in lipid droplets when treated with PER alone or in combination with PB, while PB had no significant influence on fat accumulation in these cells. Interestingly, Yang et al^34^ reported that PER stimulated triglyceride synthesis and lipids droplets while suppressing lipid oxidation in palmitic acid-induced HepG2 cell steatosis model, which is in agreement with our findings.

In conclusion, our data on the effect of PER on the liver, and on HepG2 cells in vitro, are consistent with reports stating that PER-induced toxicity under in vivo and in vitro studies, is explained through PER metabolism in the liver, that generates oxidative stress and steatohepatitis^35^.

To test if the exacerbated response to alcohol-induced liver injury in mice previously exposed to GWI-related chemicals was due to the low level protracted inflammation observed in the liver, we used PLX3397 to deplete the GWI-associated build up of macrophages prior to exposure to alcohol. PLX3397 is an oral tyrosine kinase inhibitor of CSF1R in macrophages, that was able to attenuate the negative effects of inflammation in several chronic conditions including fatty liver, obesity, diabetes and even cancer in preclinical studies^36^. PLX3397 has been approved to be used in clinical trials for various types of cancer that required depletion of macrophages with role in tumorigenesis^37^. Moreover, previous studies found that PLX3397 was also an inhibitor of PDGFb receptor, acting effectively to stop cell proliferation^38^. Since the liver injury involves persistent inflammation and activation of HSC that express PDGFb receptors with role in fibrogenesis, we tested the efficacy of PLX3397 in reducing the alcohol-induced liver damage in GWI mice. As expected, PLX3397 lowered the density of Kupffer cells and monocyte-derived macrophages, as well as pro-inflammatory cytokines in liver samples from GWI-mice treated with vehicle or EtOH. Furthermore, the marker of activated HSC, aSMA, was reduced, which resulted in less fibrosis of the liver in GWI-mice when treated with PLX3397 during EtOH binging. One of the molecular mechanisms involved in liver damage is the ER stress, which can lead to cell death, via apoptosis and autophagy in hepatocytes^39^. Remarkably, the presence of lipid droplets in livers of GWI mice treated with PLX3397 prior to EtOH, was also reduced as compared to GWI mice that received the alcohol without PLX3397. These results suggest that macrophage-derived cytokines could contribute to changes in lipid metabolism and storage in hepatocytes under alcohol-induced stress and damage.

To test the possibility that PER, PB or the combination of both substances could have a direct effect on lipid metabolism in hepatocytes, we did experiments in vitro, using HepG2 cells. The results indicated that the combination of PER and PB was more effective than each of the two components alone, favoring lipid storage inside hepatocytes, via upregulation of SREBP1 gene of *de novo* synthesis of lipids, as well as structural (e.g., PLIN2) and functional (e.g., PNPLA3) proteins of lipid droplets.

**Figure 11:**
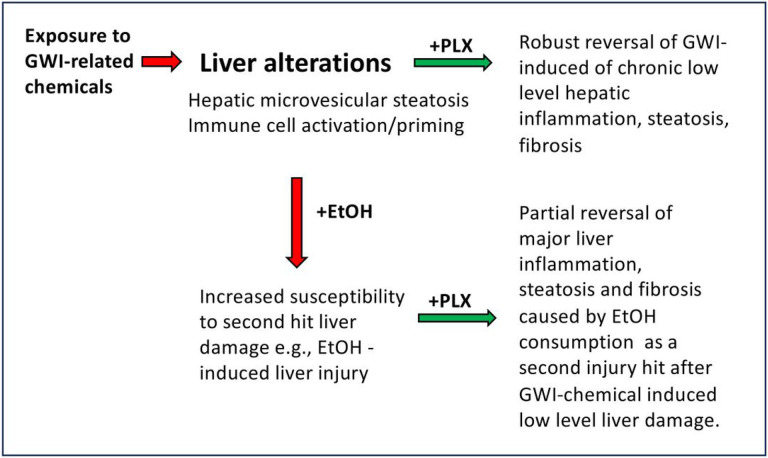
Schematic representation of critical changes in the liver in mice exposed to GWI-related substances and subsequently challenged with EtOH in the absence or presence of PLX3397 inhibitor of CSF1R of macrophages. Mice that were exposed to a combination of PER and PB followed by a period of recovery, exhibited significant levels of hepatic microsteatosis and chronic low-grade inflammation and priming of the immune cells in the liver. Indeed, GWI mice that underwent chronic single binge of EtOH (a second hit on the liver), suffered increased liver damage as compared to Naïve mice with EtOH. Treatment with PLX3397, a small molecule that depletes macrophages, reduced inflammation and microsteatosis in mice exposed to GWI compared to Naïve controls. Moreover, GWI mice treated with PLX3397 prior to EtOH binging, decreased the level of steatosis, fibrosis and ductular reaction, compared to GWI mice that did not receive PLX3397.

In conclusion, as illustrated in Fig. 11, we have demonstrated that exposure of mice to PER and PB, was conducive to liver alterations such as microvesicular steatosis and low-grade chronic inflammation which primed the liver to react to a subsequent challenge caused by alcohol binging, that resulted in abnormal inflammation, biliary hyperplasia and fibrosis. Our study also showed that using PLX3397 to deplete macrophages during alcohol binging, mitigated the hepatic inflammation and decreased alcohol-induced liver damage. Taken together, while increased incidence of overt liver disease has not yet been associated with GWI, significant changes to liver physiology may be evident resulting in an increased susceptibility of Veterans with GWI to second insults to the liver such as alcohol-induced liver injury.

## Methods

### Chemicals, kits, tissue culture media, antibodies.

PER was purchased from Chem-Service Inc (West Chester, PA). PB came from Sigma-Aldrich, part of Millipore-Sigma in US (Burlington, MA; www.sigmaaldrige.com). Vectastain, DAB Substrate/peroxidase kits, hematoxylin QS were from Vector Lab (Burlingame, CA). AST, ALT Catalyst kits from IDEXX (Westbrook, ME). Oil Red O and Sirius Red staining kits were from Nova Ultra, IHC World (Ellicott City, MD). PLX3397 was from Thermo Fisher Scientific (Waltham, MA). Antigen-unmasking solution, Cytoseal XYL mounting reagent, Prolong Gold Anti Fade with DAPI mounting solution, cell culture media and solutions including high glucose-DMEM, fetal bovine serum (FBS) and penicillin/ streptomycin were from Gibco BRL purchased through Thermo Fisher Scientific (Waltham, MA). Primary antibodies were obtained from Abcam (Cambridge, MA) unless specified otherwise. The antibody to mouse C-type lectin in F4/80-positive cells (Clec4f) was from Invitrogen by Thermo Fisher (Rockford, IL). All primers used in RT-qPCR were ordered from Qiagen (Germantown, MD). All the other chemicals were purchased from Millipore-Sigma (Burlington, MA) unless otherwise stated, and were of the highest grade available.

## Animal experiments

Adult, 8-week-old male C57BL6/J mice were purchased from The Jackson Laboratory (Bar Harbor, ME) and maintained in a temperature-controlled environment at 20-22°C with a light-dark cycle of 12:12 hour, having free access to food and drinking water. All animal procedures were performed in accord with protocols approved by the Institutional Animal Care and Use Committee of University of Texas at Austin (Austin, TX). The mice were randomly divided into two groups: Naïve mice, injected daily with vehicle (DMSO), and GWI-mice that were administered a mix of 100 mg/kg PER and 0.7 mg/kg PB for 10 days. This model has been established as a mouse model of GWI in other laboratories^21^. After 4 months of recovery, the two initial groups of mice were divided into the following groups: GWI and Naïve mice that were treated with 50 mg/kg PLX3397^40^ by gavage, every second day for 10 days; GWI and Naïve mice treated with vehicle as controls for PLX3397. The GWI and Naïve mice that received either PLX3397 or vehicle, were further treated with a chronic-plus-single-binge EtOH for 10 days, as described^41^. The mice were euthanized by intraperitoneal injection of at least 150 mg/Kg Euthasol (pentobarbital sodium plus phenytoin sodium euthanasia solution, purchased from Animal Health International, Loveland CO, USA) followed by a procedure of thoracotomy. Serum and liver samples were collected from all groups of mice. The timeline and experiment design are schematically shown in Fig. 1.

## Assessment of mRNA expression by RT-qPCR

Total RNA was isolated from frozen sample of liver tissue using RNeasy kit from Qiagen (Germantown, MD), followed by cDNA synthesis with iScript kit from Bio-Rad Life Sciences (Hercules, CA), and RT-qPCR using iTaq Universal SYBR-Green Supermix from the same company. RT^2^ qPCR Primer Assays and primers for all our RT-qPCR assays were ordered online from https://geneglobe.qiagen.com/us. The RT-qPCR assays were run using CFX96 C1000 Touch Thermal cycler and Bio-Rad CFX Maestro software from Bio-Rad (Hercules, CA). The data was analyzed as previously described^20^. Fold changes in gene expression were normalized to the housekeeping gene glyceraldehyde-3-phosphate dehydrogenase (GAPDH).

## Serum biochemistry

Aspartate- and alanine-transaminase (AST, ALT) assays were performed using Catalyst One Analyzer from IDEXX Laboratories (Westbrook, ME).

## Immunohistochemistry (IHC)

For IHC, liver paraffin sections of 4 μm were immunolabeled with primary antibodies specific to proteins of interest, and stained using VectaStain kits and counterstained with hematoxylin as previously described^23^. The IHC slides were scanned with a Leica SCN400 scanner at 20X magnification, followed by screenshots at 10X magnification. The percent stained area was measured by image analysis which was carried out using the 1.52a version of ImageJ software, as downloaded from the NIH website (http://imageJ.nih.gov/ij ).

## Fluorescence labeling and confocal microscopy of lipid droplets and apoptosis marker

Lipid droplets (LD) were stained with Oil Red O in frozen liver sections from mice. Thus, frozen liver tissue embedded in OCT medium was sectioned at 8μm using Leica Cryostat CM1850 (Leica Biosystems, www2.leicabiosystems.com), and the sections were stained using the Oil Red O kit from IHC World (Ellicott City, MD). Images of Oil-Red-stained LD were taken using a confocal laser scanning system from Leica Microsystems Inc. (Buffalo Grove, IL). The percent stained area was measured by image analysis as described for IHC. In HepG2 cells in vitro, LD were stained with Nile Red as described^42^ and quantified using the ImageJ software. Additionally, specific staining for apoptosis, was performed on HepG2 cells, using the Apoptosis/Necrosis assay kit from Abcam (Cambridge, MA).

## Liver histopathology, ductular reaction and fibrosis

Hematoxylin and eosin (H&E) staining was performed on 4 μm sections of paraffin-embedded livers, as previously described^20^. Expression LD-associated genes encoding for perilipin 2 (PLIN2) and patatin-like phospholipase domain-containing 3 (PNPLA3) were measured by RT-qPCR. Biliary hyperplasia was assessed measuring expression of cytokeratin 19 (CK19) marker of cholangiocytes at mRNA and protein level, using RT-qPCR and immunohistochemistry (IHC) staining, respectively. The expression of fibrosis-related genes such as connective tissue growth factor (CTGF), platelet-derived growth factor beta (PDGF b) and transforming growth factor beta-1 (TGF-b1) was assessed by RT-qPCR of liver samples. Collagen 1A1 (Col1A1) and other extracellular matrix proteins associated with fibrosis were measured using Sirius Red staining. All these methods were applied according to the manufacturers’ instructions unless otherwise specified.

## Assessment of hepatic inflammation

Changes in density of Kupffer cells and monocyte-derived macrophages recruited to the liver were detected by RT-qPCR and/or IHC for Clec4f and CD11b, respectively. Hepatic inflammatory cytokines interleukin-1 beta (IL-1b), interleukin-6 (IL-6), C-C motif chemokine ligand 2 (CCL2), tumor necrosis factor-alpha (TNF-a) were assayed by RT-qPCR and ELISA.

## Statistics

Quantifications of data from RT-qPCR, ELISA or image analyses were done calculating the average and standard error of the mean (SEM) of three replicates for each group of tested animals. For in vivo experiments, the number of animals (N) in each treatment group was 4, or otherwise specified in the Results section for each experiment. The statistical differences between two groups, was calculated by Student’s T-test, and were marked as significant when the p value was less than 0.05.

## Data Availability

The datasets generated and analyzed during the current study are available from the corresponding author on reasonable request.
